# Challenge and threat motivation: effects on superficial and elaborative information processing

**DOI:** 10.3389/fpsyg.2014.01170

**Published:** 2014-10-14

**Authors:** Ricardo Fonseca, James Blascovich, Teresa Garcia-Marques

**Affiliations:** ^1^ISPA – Instituto UniversitárioLisboa, Portugal; ^2^University of CaliforniaSanta Barbara, CA, USA

**Keywords:** biopsychosocial model, dual-processing, persuasion, challenge, threat

## Abstract

This paper integrates the motivational states of challenge and threat within a dual processing perspective. Previous research has demonstrated that individuals experience a challenge state when individuals have sufficient resources to cope with the demands of a task (Blascovich et al., 1993). Because the experience of resource availability has been shown to be associated with superficial processing (Garcia-Marques and Mackie, 2007), we tested the hypothesis that challenge is associated with superficial processing in two persuasion experiments. Experiment 1 revealed that inducing attitudes of participants in a challenge state was not sensitive to the quality of arguments presented. Experiment 2 demonstrated that the effect occurs even when task engagement, manipulated by the presence (vs. the absence) of a task observer (Blascovich et al., 1993), is high. The implications of these results for the biopsychosocial model model and the cognitive and motivational literature are discussed.

## INTRODUCTION

The biopsychosocial model (BPS) of challenge and threat (Blascovich, 2008; Blascovich and Mendes, 2010) is a well-validated theoretical model in the social motivation literature. Hypotheses based on the BPS are typically examined via neuroendocrine-driven patterns of cardiovascular (CV) responses (Blascovich, 2008) derived from Dienstbier’s (1989) neuroendocrine model of “physiological toughness.” The empirical validation of these CV patterns in humans is based on correlational and experimental studies (e.g., Tomaka et al., 1993, 1997) as well as on substantial convergent validational studies spanning a wide range of social psychological phenomena, including stigma (Blascovich et al., 2001; Mendes et al., 2002; Vick et al., 2008), social facilitation (Blascovich et al., 1999), attitude functionality (Fazio et al., 1992; Blascovich et al., 1993), social comparison (Mendes et al., 2001), etc.

Empirically, challenge in contrast to threat, as indexed cardiovascularly, has been nearly exclusively linked to better performance (e.g., Tomaka et al., 1993; Blascovich et al., 2004; Seery et al., 2004; Mendes et al., 2007). However, that is not always the case. At least one investigation has linked challenge to poorer performance compared to threat. Specifically, Hunter (2001) found that a signal detection task that required close attentional processes was performed better by threatened than by challenged participants.

In this paper, we address the question of whether this “discrepancy” is caused by differences in the depth of cognitive processing associated with challenge and threat. Specifically, we raise the hypothesis that challenge may lead to poorer performance when more analytic processes are required. By raising this novel hypothesis, we establish a relationship between the motivational states of challenge and threat and dualistic modes of information processing (contrasting analytic/central processing with non-analytic/superficial processing). Specifically, we predicted that challenge states, relative to threat states, would elicit more superficial information processing.

### THE BIOPSYCHOSOCIAL MODEL OF CHALLENGE AND THREAT AND COGNITIVE PROCESSING

Theoretically, “pure” challenge and threat are endpoints of a biopolar approach/avoidance continuum activated in motivated performance situations (i.e., situations that are goal-oriented, self-relevant to the individual, and task engaging; for reviews, see Blascovich and Seery, 2006; Blascovich, 2008). Relatively speaking, challenge refers to generally affectively positive, approach–approach motivation, whereas threat refers to generally affectively negative approach–avoidance motivation (Blascovich and Mendes, 2010).

The activation of challenge or threat, and the subsequent biological, emotional, behavioral and cognitive responses, results from relative evaluations of personal resources (for example, abilities, skills, previous knowledge) and task demands (for example required effort, uncertainty, familiarity; see Blascovich, 2008; Blascovich and Mendes, 2010). When individuals implicitly and/or explicitly evaluate resources as sufficient or exceeding their evaluations of the task demands necessary to address the motivated performance situation, challenge results. When individuals implicitly or explicitly evaluate fewer resources than those needed to address the situation, threat results.

Therefore, challenge is defined psychologically as a mental state in which the individual perceives himself or herself as being able to cope with a task and threat is defined by a mental state in which the individual perceives himself or herself as unable to cope with a task.

Hence, the challenge-threat evaluation process can be either automatic or deliberate or both and is driven by several intra- and interpersonal factors (e.g., self-esteem, familiarity) that can affect resources and demands simultaneously. For example, familiarity can lead to challenge because it increases evaluation of resources (via previous knowledge available) or decreases demands (via decreases in task difficulty) or both, the opposite being true for threat (Fazio et al., 1992; Blascovich et al., 1993; Mendes et al., 2002).

In terms of CV activation, both challenge and threat induce increases in ventricular contractility (VC, i.e., the strength of contractions of the left ventricle of the heart) as well as heart rate (HR) from resting baseline levels in active coping situations (those requiring task engagement and instrumental cognitive responses; cf Obrist, 1981; Blascovich, 2008). These responses result from increased sympathetic neural and adrenal medullary (SAM) axis activation and index task engagement. The former, VC, is a stronger predictor than HR (see Blascovich et al., 2002; Blascovich and Seery, 2006; Blascovich, 2008) most likely because VC is controlled purely sympathetically whereas HR is only partially regulated sympathetically.

When the evaluation of resources and demands results in challenge, SAM axis activation also increases the release of epinephrine into the bloodstream, resulting in decreases in total systemic peripheral vascular resistance (TPR, i.e., a measure of the resistance of the arteries) and increases in cardiac output (CO, i.e., amount of blood pumped by the heart on any given beat and expressed in beats per minute). However, in addition to activating the SAM axis, threat triggers the hypothalamic pituitary adrenal (HPA) axis, resulting in the release of cortisol into the bloodstream, which counteracts the SAM effects on TPR and CO and results in little change or even increases in TPR and decreases or no changes in CO (Blascovich, 2008).

In terms of task performance during challenge and threat, research is not completely consistent. Although challenge typically results in better performance, poorer performance occurs on occasion. Examples of tasks in which performance is better during challenge are myriad; for example, better Trier-type math performance (Tomaka et al., 1993), faster pairwise preference decisions (Fazio et al., 1992; Blascovich et al., 1993), greater accuracy for object and pattern recognition judgments (Blascovich et al., 1999), and better scores on the Remote Associates Task (Seery et al., 2004; for reviews see Blascovich, 2008).

Some studies, however, demonstrate that challenge is associated with poorer performance, especially when more controlled (i.e., thorough, exhaustive) processing of information is required. For example, in Hunter’s (2001) investigation, participants were asked, prior to a criterion task, to either read (challenge condition) or sing (threat condition) the first two stanzas of the U.S. national anthem as a warm up to anticipating a latter phase of the experiment in which they would be required to read or sing all of it while being video- and audio-recorded. The interim criterion task required participants to view pairs of words and read some of them aloud. By the end of the presentation, participants were asked to recall all the words they saw and identify intrusions among a list of words. Results revealed that those who were threatened by the possibility of having to sing the national anthem exhibited the BPS CV pattern consistent with threat, but recalling more words correctly and identifying more intrusions correctly, than those who exhibited the BPS CV challenge pattern. Feinberg and Aiello’s (2009, Experiment 1) investigation found similar results. They asked participants to perform a mental arithmetic task that consisted of summing or subtracting a series of numbers that were presented briefly (1 s) on the computer screen, the authors observed that the participants who were challenged made more mistakes than those who were threatened.

To date, few explanations and discussions regarding these variations in results appear in the literature. However, one conclusion that is clear from the studies described above is that the types of tasks and the levels and amounts of cognitive processing required may not be equivalent across the investigations. Of course, it is difficult to estimate the exact level and type of cognitive resources involved in each task, but it seems likely there are such differences.

It is proposed here that challenge is likely associated with more superficial mode information processing under certain circumstances. By definition, superficial processing is an effortless type of cognitive processing in which individuals do not spend much time or mental effort in generating a response (see Chaiken and Trope, 1999). Compared to systematic elaborative processing (see Chaiken and Trope, 1999), which is a more effortful type of cognitive processing, superficial processing is an alternative route to achieving a response that demands less capacity and motivational resources.

In fact, some BPS evidence models suggest this. For example, in the Blascovich et al. (1993) experiment, participants were asked to observe a series of novel (to participants) abstract paintings while the appropriate CV measures were recorded. Participants were instructed to vocalize whether and how much they liked or disliked each painting using a four-point evaluative scale. On a subsequent task, all participants had to make a rapid pairwise preference decision (i.e., within 2.5 s) regarding which of two paintings presented side-by-side that they preferred. For half of the participants, the paintings presented in the pairwise preference task were those that they had previously seen individually (i.e., paintings familiar to them and for which they had already formed an attitude). For the others, the paired paintings were novel. Participants in the familiar condition evidenced CV challenge physiologically, while those in the non-familiar condition evidenced CV threat.

These data are consistent with the hypothesis that challenge is associated with more superficial processing because participants were able to use the available response they had “rehearsed” on each painting instead of computing a new one; this is a typical indication of more automatic processing (Reder and Ritter, 1992; Smith, 1994; Garcia-Marques, 1999). In fact, evidence in the cognitive and social cognitive literature has explicitly supported the notion that an experience of resource availability (i.e., when a response is available and accessible in memory), is associated with superficial processing (e.g., Garcia-Marques and Mackie, 2001, 2007). This becomes more relevant if we consider that a challenge state occurs when individuals assess his/her resources as “sufficient to meet task demands.” Thus even when individuals do possess the sufficient resources to engage in deeper processing they will often fail do so.

### IMPLICATIONS OF A DUAL-PROCESS APPROACH TO CHALLENGE AND THREAT

Testing the hypothesis that challenge is associated with superficial processing requires examination of the effect of challenge/threat motivation on the depth of information processing. One useful framework is provided in the persuasion literature one of the most well tested empirical arenas regarding the dualistic nature of information processing. In this literature, superficial processing has been associated with a specific pattern of response to persuasive arguments. For decades now, research (Petty and Cacioppo, 1986; Petty and Wegener, 1998; Chen and Chaiken, 1999 see also Petty and Briñol, 2011) clearly has demonstrated that for individuals to react differentially to argument quality (i.e., strong and weak), they have to be sufficiently task engaged to process information analytically, whereas those who process information superficially are not differentially affected by strong and weak arguments. Thus, regarding persuasion, task engagement increases the probability of deeper processing *per se* (Petty and Cacioppo, 1986: Chen et al., 1996). According to the BPS of challenge and threat (Blascovich, 2008), if task engagement (a function of the personal relevance of the task) does not occur, then challenge and threat cannot be differentiated physiologically (as demonstrated by Tomaka et al., 1993). Therefore, it is possible that if the level of task engagement needed to promote physiological differentiation engenders deeper processing unilaterally, then challenge and threat will promote similar processing effects. However, if this differentiation occurs with task engagement at a moderate level, it may allow precursors of depth of processing as defined by the BPS model and (see Garcia-Marques et al., 2013 for a review), which causes challenge to be associated with more superficial processing as previously argued.

In the experiments described below, we tested the general hypothesis that challenge relative to threat is associated with more superficial processing of persuasive messages (Experiment 1). We subsequently undertook a more direct approach to the examination of task engagement in the relationship between challenge and information processing depth (Experiment 2).

## EXPERIMENT 1

To test our general hypothesis, perceptions of task demands were manipulated. According to the BPS model, if a task is not demanding, individuals will be more likely to evaluate sufficient resources to cope with the task and will likely be motivationally challenged. If a task is demanding, individuals will be more likely to evaluate less of this sufficiency and will be more likely to be motivationally threatened.

The experimental tasks here were designed to induce either challenge or threat without compromising participants’ capacity to process information during the task. Research within the dual process models of information processing (e.g., Petty and Cacioppo, 1986; Eagly and Chaiken, 1993; Petty and Wegener, 1998) has demonstrated that the depletion of cognitive resources—for example, due to cognitive distractions or cognitive fatigue—reduces the probability of cognitive elaboration. Based on this reasoning, a visual acuity game task was created. Participants performed either a difficult or easy version of the game, long enough to promote task engagement and induce the corresponding challenge and threat states. Next, a strong or weak persuasion message was presented immediately after the visual game to observe the effect of challenge on information processing. It was expected that task engagement, and the corresponding CV patterns, would carry over to the persuasion task and that challenged compared to threatened participants would not differentiate strong from weak arguments in terms of attitude change, which suggests less processing in this condition.

### METHOD

#### Participants and design

Fifty-two UCSB undergraduates (31 males; Mean age = 19.2, SD = 1.22), who received course credit for participation, were randomly assigned to conditions in a 2 (demanding vs. non-demanding game) × 2 (strong vs. weak arguments) between-subjects factorial design.

#### Procedure^1^

***Pre-attitudes and baseline recordings***. Participants arrived individually at the lab and, read and signed a consent form. Next, they completed an initial questionnaire in which they reported their attitudes toward several topics, including the one that was subsequently the target of persuasion (with a simple item stating the agreement with the attitude subject “to impose restrictions on industry to minimize the effects of acid rain”). Next, the experimenter attached appropriate physiological sensors to participants, and a 5 min baseline rest period began during which the experimenter left the room. Subsequently, the experimenter returned and instructed participants that the goal of the experiment was to investigate individuals’ visual abilities on different tasks while being monitored physiologically. The experimenter initiated the experiment via computer, restarted the physiological recordings and sat on the opposite side of the room out of view of participants for the entire session.

Physiological signals were recorded using a Biopac impedance cardiograph (Model NICO100C), a NIBP100A blood pressure monitor and a Biopac electrocardiograph amplifier (Model ECG100C). Electrocardiographic (ECG) and impedance cardiographic (ZKG) recordings provided continuous measures of cardiac performance. Employing a tetrapolar aluminum/mylar tape electrode system, impedance cardiography provides basal transthoracic impedance (Z0) and the first derivative of basal impedance (dZ/dt). Two pairs of ZKG tape electrodes were fastened around the participants’ necks and torsos. A 400 ìA AC 50 kHz current is passed through the top and bottom electrodes, and basal impedance is measured via the inside electrodes. ECG recordings were attained using a modified lead II configuration (lower left torso and upper right torso, with impedance cardiography providing an internal ground). Continuous, non-invasive blood pressure measurements were obtained using a NIBP100A blood pressure monitor that included a pressure sensor placed on the wrist over the radial artery. This device operates via a “sweep technique,” that applies varying force on the radial artery. The counter-pressure in the artery produces a signal, which is digitized and used to calculate blood pressure parameters. Finally, data were integrated with an MP150 and displayed and stored with Acknowledge software (Biopac, Goleta, CA, USA). Mindware software was used to edit artifacts and organize and score the data.

***Induction of the motivational states***. For the first task, instructions presented via a computer monitor informed participants that they were to play a visual ability game. Their task was to observe and compare several pairs of geometrical shapes (triangles, squares, circles) of different sizes and decide which of the each pair was larger. Using a computer mouse to respond, each participant performed 30 trials successively and in random order. Those in the non-demanding condition performed 15 standard trials, in which the difference between the two shapes was somewhat easy to discriminate (1 cm), and 15 non-demanding trials, where the difference was quite obvious and even easier to discriminate (1.5 cm). Those in the demanding condition performed 15 standard trials and 15 demanding trials, where the difference was not obvious and was difficult to discriminate (0.5 cm). Standard trials were added so that the demanding version would not be extremely difficult and the non-demanding version would not be extremely easy. To increase task engagement, participants were also informed that they had only 3 s to make each of their decisions and that most people could respond correctly within that interval and instructed that they should pay attention carefully to each pair of shapes. This task had been pre-tested at different phases. First we insured that participants perceived each task differently by measuring perceived task difficulty (being significantly greater in the threat condition). Subsequently, 21 participants performed the game while their physiological responses were recorded, insuring the presence of the physiological pattern associated with each motivational state (see Fonseca, Unpublished doctoral dissertation).

***Experimental task***. Immediately following the 30 trials, participants were told that they would continue the game later but in the interim were instructed to read a (strong or weak) message arguing against imposing restrictions on industry to minimize the effects of acid rain (adapted from Garcia-Marques and Mackie, 2001). Using a 1 (total disagreement)–7 (total agreement) scale, participants rated how much they agreed with three statement related to the topic: “*The government should impose controls on industry to help minimize the effect of acid rain in the US”; “Increases in problems with acid rain in the US should not be blamed on the activities of industries operating in affected areas”; “The government should require the installation of sulfur dioxide emissions control devices in factories operating in the US”.*

***Conclusion of the game and debriefing***. Subsequent instructions informed participants that they would continue the game for 15 more standard trials. At the end, they received bogus feedback on their performance. They were told that their performance was between 80 and 100% accurate. Next, they were told that the experiment was over. The experimenter, then, turned off the physiological recordings, removed the physiological sensors and asked participants to complete a final questionnaire that assessed relevant variables, such as perceptions of task difficulty. Subsequently, all participants were fully debriefed and thanked for their participation.

#### Measures

***Challenge and threat indexes***. Mean values of HR, VC, CO, and TPR were calculated for each minute of the baseline, the game, and the persuasion task. Reactivity scores for each CV response were then calculated for the game task and the persuasion task by subtracting the fifth minute of the baseline from the first minute of the game task and from the first minute of the persuasion task, in accordance with well-established procedures in the BPS model literature.

***Attitude-change index***. A composite measure based on the average of each participant’s response on the three items regarding the acid rain message (64% of the total variance explained; Cronbach’s α = 0.72) was subtracted from the pre-measure, providing the attitude-change index.

### RESULTS AND DISCUSSION

#### Effectiveness of the task demands manipulation

The effectiveness of the manipulation of task demands on the challenge and threat evaluations was confirmed by participants’ ratings on the item “*In general, I think I had sufficient capacity to handle what was demanded by the game.”* These ratings were analyzed via a one-way analysis of variance (ANOVA). Although participants in the non-demanding condition reported different (more) capacity to deal with handle task demands (*M* = 5.64, SD = 1.62) than participants in the demanding condition (*M* = 4.76, SD = 1.56), the mean differences did not reach conventional levels of statistical significance: *F*(1,48) = 3.79, *p*= 0.057, η^2^ = 0.08.

#### Challenge and threat data: baseline differences

No baseline differences emerged across the experimental conditions when the fifth minute of baseline HR, VC, CO, and TPR values were entered simultaneously as dependent measures in a 2 (demanding vs. non-demanding game) × 2 (strong vs. weak message) MANOVA [version game: *F*(4,39) = 1.60, *p* = 0.194, message quality, *F* < 1]. Additionally, the interaction did not reach significance levels, *F*(4,39) = 2.02, *p* = 0.110.

#### Task engagement

The hypothesis that participants were task engaged while playing the game was confirmed via independent *t*-tests on VC reactivity indexes and also on HR. As expected, VC was significantly different (greater) than zero, VC, *t*(51) = 2.56, *p*= 0.006, *d*= 0.71 (*M*= 1.87, SD = 5.28). We also found that the HR was significantly different (greater) than zero: *t*(51) = 3.23, *p*= 0.001, *d*= 0.90 (*M* = 12.77, SD = 28.52). Additionally, as expected, task engagement continued during the persuasion task, as indicated by the significant differences from zero: VC, *t*(51) = 1.98, *p* = 0.053, *d*= 0.55 (*M*= 1.19, SD = 4.36) and HR, *t*(51) = 3.82, *p* < 0.001, *d*= 1.07 (*M* = 12.86, SD = 24.25).

#### Challenge and threat indexes

The hypothesis that the non-demanding version of the game resulted in a CV challenge pattern was tested by entering the CO and TPR reactivity indexes in two separate one-way ANOVAs with the game version as the fixed factor. The main effect of game version emerged on both measures: CO *F*(1,50) = 4.29, *p* = 0.043, η^2^ = 0.10; TPR *F*(1,50) = 5.47, *p*= 0.023, η^2^ = 0.12. As expected, participants in the non-demanding game condition, compared to those in the demanding condition, exhibited a challenge pattern mapped by increases from the last minute of the baseline in CO (Mnon-demanding = 0.62, SD = 0.48 vs. Mdemanding = -0.79, SD = 0.49) and decreases in TPR (Mnon-demanding = -107.71, SD = 51.14 vs. Mdemanding = 61.46, SD = 25.60). Additional analyses revealed that these patterns persisted during the persuasion task: those who performed the non-demanding version remained challenged while processing the persuasive message, compared to those who performed the demanding game [CO, Mnon-demanding = 1.22, SD = 0.88 vs. CO, Mdemanding = -1.48, SD = 0.92, *F*(1,48) = 4.36, *p*= 0.042, η^2^ = 0.10; TPR, Mnon-demanding = -92.30, SD = 65.75 vs. Mdemanding = 92.01, SD = 61.48, *F*(1,48) = 3.93, *p* = 0.053, η^2^ = 0.09]^2^.

#### Attitude change data

**FIGURE 1:**
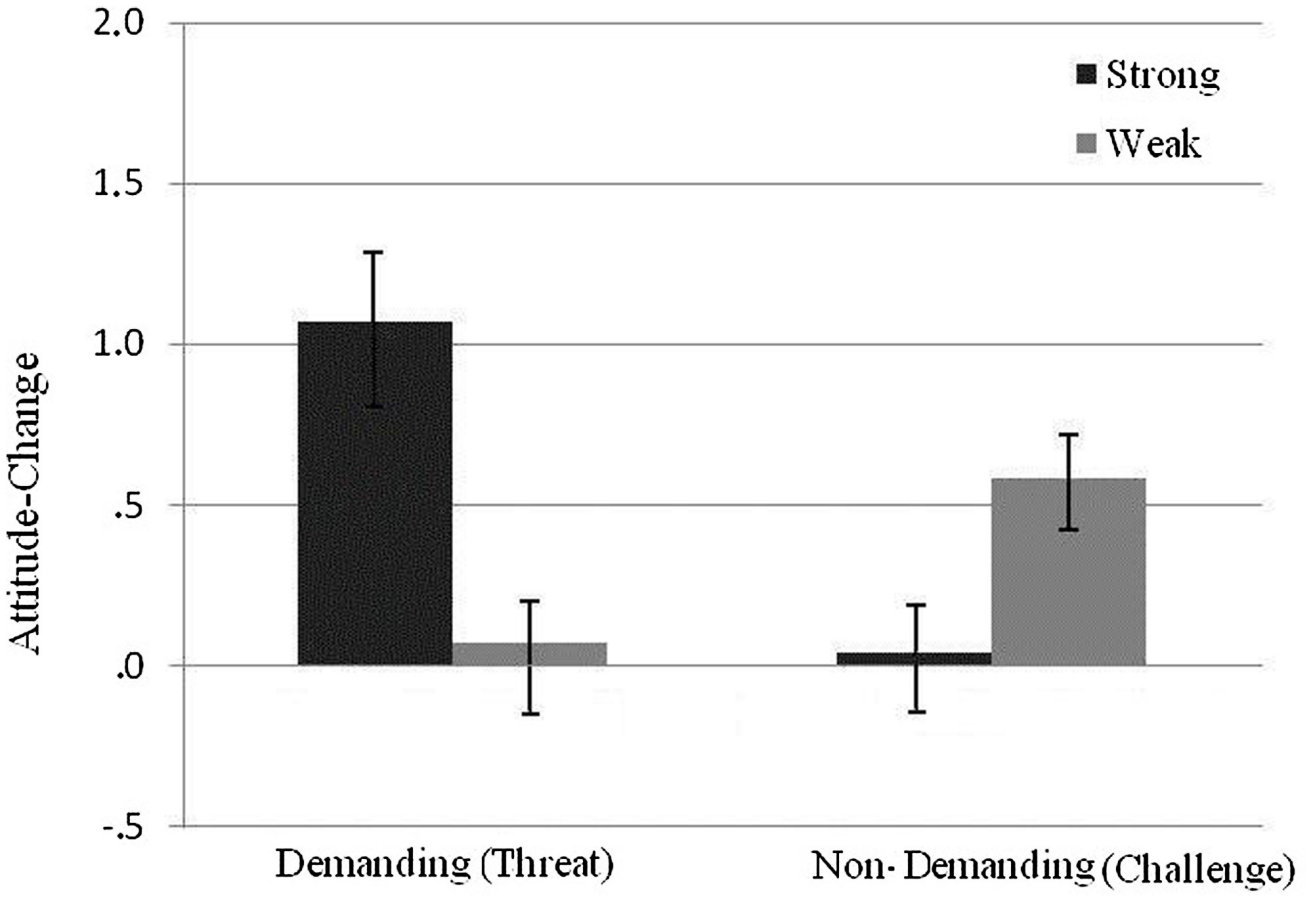
**Index of attitude change toward the target issue as a function of demands and message quality**. Error bars represent standard errors of the mean.

The general hypothesis was that challenge would be associated with superficial processing. As such, it was predicted that those who were challenged while performing the non-demanding version of the game beforehand would exhibit attitude changes that did not reflect differentiation between strong and weak arguments. This hypothesis was supported when the acid rain attitude change index was entered as a dependent measure in a 2 (demanding vs. non-demanding game) × 2 (strong vs. weak message) ANOVA and an interaction emerged: *F*(1,48) = 4.11, *p*= 0.048, η^2^ = 0.08. As hypothesized, this interaction was driven by the differential impact of strong and weak arguments on attitude change manifested in the demanding (threat) condition but not in the non-demanding (challenge) condition (see **Figure 1**). However, if it is clear that those who were challenged reacted equally to strong and weak arguments, *t*(48) = -1.06, *p*= 0.296, *d* = 0.31, it is less clear that those who were threatened were more persuaded by the strong than by the weak message as the two-tailed contrast did not reach the standard levels of significance, *t*(48) = 1.81, *p*= 0.076, *d* = 0.52. No main effect of the version of the game or the message quality was found (*F’s* < 1).

Together, these results support the general hypothesis that challenge, as defined and measured according to the BPS model, is associated with the automatic processing of tasks that require cognitive elaboration of information. In addition to replicating other results in the BPS literature (Blascovich et al., 1993; Hunter, 2001), this is some of the first evidence to examine challenge and threat in terms of cognitive mechanisms, specifically within a dual processing framework. Additionally, the results suggest that the overarching task engagement necessary to interpret BPS patterns of activation is similar to a moderate level of motivation in the persuasion literature. Specifically, if the necessary task engagement specified in the BPS model was related to the high motivation level (that persuasion models, associate with increased depth of cognitive processing, Petty et al., 1981), both challenge and threat states should have led to thorough processing. The data here suggest that careful processing is true only for threat and not for challenge. Given the moderated motivational level, it was possible to identify the effect of manipulations of other factors, such as familiarity (see Claypool et al., 2004).

An alternative possibility is that the manipulation of challenge and threat states itself affects the level of motivation and engagement in the task. For some reason, our instructional manipulations might have been sufficient for increasing HR and VC in both conditions but only sufficient for increasing analytic processing in the threat condition. Thus, in Experiment 2, we added a task engagement manipulation to the experimental paradigm used in Experiment 1, expecting that if the effects of challenge and threat on processing were independent of intensified task engagement, then our interpretation would be strengthened.

## EXPERIMENT 2

Experiment 2 closely replicated Experiment 1, but we added a manipulation of task engagement via the presence or absence of observers. This addition was based on Blascovich et al. (1999) studies that demonstrate that the effect of a variable such as familiarity on challenge is moderated by a factor that increases individuals’ task engagement. As in Experiment 1, challenge and threat were induced via the perception of task demands. Specifically, challenge and threat were induced by having participants accompanied, or not, by an observer while performing either a non-demanding or a demanding version of the visual ability task.

Immediately after inducing the motivational states, a strong or weak persuasive message was presented to allow us to test the predicted effects. Our hypothesis was that if task engagement increases motivation to process, then challenge and threat should both increase analytic processing; a result opposite to what was found in Experiment 1. However, if task engagement does not necessarily increase motivation to process, then challenge should lead to superficial processing independently of a condition where task engagement is intensified (i.e., in the presence of others). Additionally, because challenge and threat are activated only when task engagement is reached (as shown by significant increases in HR and VC), we expected that in the low engagement condition (i.e., the alone condition), challenge and threat would not be differentiated; thus, neither one of them would affect information processing.

### METHOD

#### Participants and design

Ninety-eight UCSB undergraduate students (55 females; Mage = 19.18, SD = 1.32) received course credit for their participation. Participants were randomly assigned to a 2 (demanding vs. non-demanding game) × 2 (strong vs. weak message) × 2 (alone vs. presence of observer) between-subjects factorial design.

#### Procedure

This experiment was a close replication of Experiment 1. First, participants arrived, were greeted, and read and signed the consent form. Next, they filled out the initial questionnaire and reported their attitudes toward several attitudinal issues, including one about acid rain, which served as the pre-attitude measure. Subsequently, appropriate sensors and transducers were attached to participants, and a 5 min baseline of CV recording commenced.

The experimenter then returned to the testing room and stopped the recording of the physiological data. For those in the presence condition, the experimenter returned accompanied by a male or female confederate (matching participant’s gender) who sat in the room in which the experiment took place within the participants’ visual field on the opposite side of the desk at a distance of 1.5 m from the participant. Similar to Blascovich et al.’s (1999) investigation, the confederate was introduced as a visitor from another laboratory interested in how people perform tasks on a computer while being connected to physiological equipment, and for that reason he/she would stay in the room and observe the performance.

The experimenter then commenced recording the physiological data and left the room. Instructions on the computer screen informed participants about the general goals of the experiment and how to perform both the visual ability game and the persuasion task. Initially, participants performed 30 trials of a randomly assigned demanding or non-demanding version of the game. Then, they read the strong or weak acid rain message for which they rated their opinion in three items anchored in a 7-point scale.

Upon completion of both tasks, the experimenter returned to the room, removed the physiological sensors from participants and handed them the control measure questionnaire. Finally, all participants were thoroughly debriefed and thanked for their participation.

### MEASURES

**Attitude change measure**. As in Experiment 1, a composite post-attitude measure about the acid rain (explaining more than 80% of the total variance; Cronbach’s α = 0.87) was subtracted from the pre-attitude measure, providing our attitude-change index.

**Challenge and threat indexes**. As in Experiment 1, reactivity scores for each CV response were calculated by subtracting the fifth minute of the baseline by the first minute of the game task and by the first minute of the persuasion task.

### RESULTS AND DISCUSSION

#### Effectiveness of the task demands manipulation

Replicating the results of Experiment 1, participants in the non-demanding condition exhibited cognitive evaluations consistent with challenge: They reported more capacity to handle the task demands (*M* = 6.13, SD = 0.21) than participants in the demanding condition [*M* = 4.97, SD = 0.20; *F*(1,91) = 16.12, *p*< 0.001, η^2^ = 0.21]. No other main effects or interaction emerged in our 2 (demanding vs. non-demanding game) × 2 (alone vs. presence of observer) ANOVA model, *Fs* < 1.

#### Challenge and threat data baseline differences

No baseline differences across the experimental conditions emerged when the fifth minute of HR, VC, and TPR values were entered simultaneously as dependent measures in a 2 (demanding vs. non-demanding game) × 2 (strong vs. weak message) × 2 (alone vs. presence of observer) MANOVA, *F’s*< 1. However, the results of the one way analysis revealed that CO was significantly different in the presence condition, *F*(1,90) = 4.55, *p*= 0.036, η^2^ = 0.10, in which alone participants exhibited greater levels of CO (*M* = 5.65, SD = 0.29) than accompanied participants (*M* = 4.78, SD = 0.29), even though there were no procedural differences at that point in time in experimental protocol. To control for these differences, CO baseline values were entered as a covariate in the relevant subsequent analyses.

#### Task engagement

As expected, the presence of an observer increased the intensity of task engagement, as confirmed by results of *t*-tests, which demonstrated that VC reactivity scores differed from zero in the presence condition, *t*(49) = 2.17, *p*= 0.034, *d*= 0.62 (*M*= 6.52, SD = 23.87), but not in the alone condition, VC *t*< 1. Additionally, although it is clear that HR reactivity scores differed from zero in the presence condition, *t*(49) = 3.16, *p*= 0.003, *d*= 0.90 (*M* = 10.24, SD = 22.89) in the alone condition, only the one-tailed test that directly tests that *M*= 5.06 (SD = 18.15) is greater than zero is significant: *t*(47) = 1.93, *p*= 0.053, *d*= 0.56. Because task engagement is a necessary condition for the activation of challenge and threat, we further tested for differences between the baseline and task periods of increases in VC and supplementarily in HR. Alone condition individuals showed no evidence of an increase in task engagement VC, *t*< 1; data regarding HR reactivity scores was only marginally significant, *t*(47) = 1.95, *p*= 0.056, *d* = 0.57 (*M* = 4.94, SD = 17.54). More importantly, as predicted, for those in the presence condition, task engagement carried over to the persuasion task, as indicated by the significant differences from baseline for VC, *t*(49) = 2.05, *p*= 0.045, *d*= 0.55 (*M*= 7.33, SD = 24.15) and corroborated by HR measures, *t*(49) = 2.35, *p* = 0.022, *d*= 0.67 (*M* = 5.92, SD = 17.87).

#### Challenge and threat indexes

Because task engagement is a necessary condition for the activation of challenge and threat states, and task engagement did not occur in the alone conditions, challenge and threat indexes were only analyzed in the presence conditions. Corroborating previous results, a significant main effect of the non-demanding version of the game emerged when TPR was entered as dependent measures in a one-way ANOVA, *F*(1,47) = 5.03, *p* = 0.029, η^2^ = 0.10. Results were not also clear with regard the CO, which revealed only a marginally significant effect, *F*(1,47) = 3.65, *p* = 0.062, η^2^ = 0.07. However, the pattern of results is consistent with what should be expected, since a challenge pattern emerged in the non-demanding version of the game, which promoted increases in CO (Mnon-demanding = 0.92, SD = 0.44 vs. Mdemanding = -0.26, SD = 0.42) and decreases in TPR (Mnon-demanding = -331.194, SD = 165.47 vs. Mdemanding = 193.45, SD = 158.72). Additional analyses confirmed that that these patterns were maintained during the persuasion task: those who performed the non-demanding version remained more challenged while processing the persuasive message than those who performed the demanding version [a pattern of results always clear for TPR, Mnon-demanding = -90.29, SD = 195.22 vs. Mdemanding = 489.08, SD = 185.52, *F*(1,46) = 4.41, *p*= 0.041, η^2^ = 0.09 and less clear for CO because the difference between Mnon-demanding = 1.62, SD = 0.53 vs. Mdemanding = 0.13, SD = 0.51, did not reach standard levels of significance, *F*(1,46) = 3.87, *p* = 0.055, η^2^ = 0.08]. No other main effect or interaction was found: message quality: CO, *F*(1,46) = 1.48, *p*= 0.229, TPR, *F*(1,46) = 1.66, *p*= 0.204; message quality X task demands interaction: CO, *F*(1,46) = 1.06, *p*= 0.308; TPR, *F*< 1.

#### Attitude change data

If task engagement engenders greater motivation to process information, then challenge and threat should both increase analytic processing, but if not, then, based on Experiment 1 results, challenge should lead to superficial processing independently of a condition where task engagement is intensified (i.e., in the presence of others). Using the acid rain attitude change index as a dependent measure, the ANOVA model defined by 2 (demanding vs. non-demanding game) × 2 (strong vs. weak message) × 2 (alone vs. presence of observer) design revealed the presence of a three way interaction, *F*(1,90) = 4.87, *p* = 0.029, η^2^ = 0.05.

The pattern of means associated with this interaction implies that challenge and threat conditions did not promote the same type of processing and that presence moderated the pattern of results found in Experiment 1. To further test this implication, we performed a separate analysis of the data for the presence and alone conditions. In the presence condition, we find the same pattern obtained in Experiment 1 defined only by a demands × message quality interaction, *F*(1,46) = 6.51, *p*= 0.014, η^2^ = 0.22.

**FIGURE 2:**
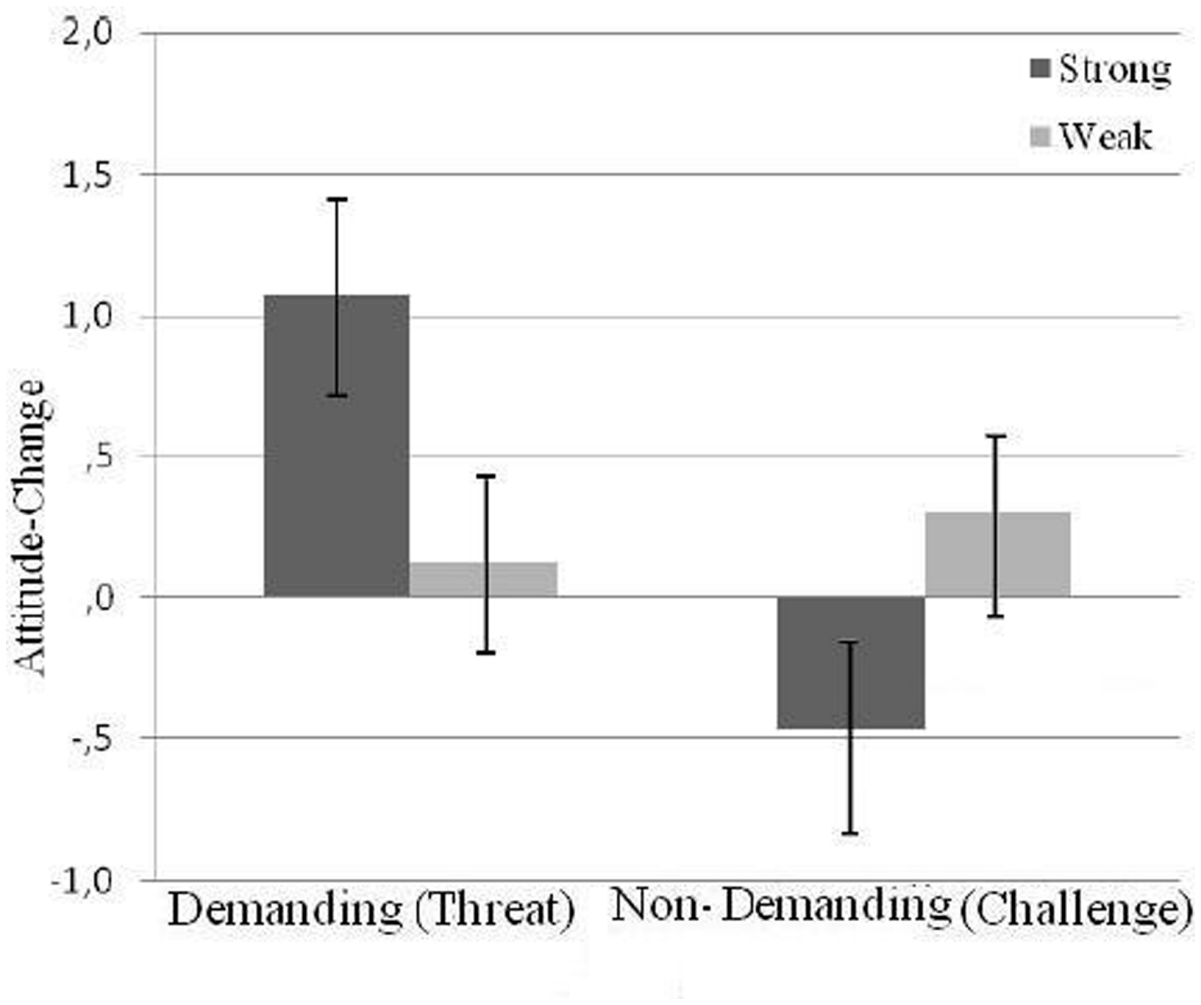
**Index of attitude change toward the target issue as a function of motivational state and argument strength for the presence condition**.

In the presence condition, we find the same pattern obtained in Experiment 1 defined only by a demands × message quality interaction, *F*(1,46) = 6.51, *p*= 0.014, h2 = 0.22. Again, the pattern of means (see **Figure 2**) go in the expected direction, suggesting differential effect of strong and weak arguments on attitude change was more clear manifested in the demanding (threat) condition. *Post hoc* analysis of that difference *t*(26) = 2.05, corresponds to a *p*= 0.050, and to a *d*= 0.80, which technically do not reach conventional levels of significance. The same analysis in the non-demanding (challenge) condition, results in an effect of even lesser magnitude *t*(24) = 1.58, *p*= 0.127, *d*= 0.62. No interaction or main effects of message quality or game demands were found in the alone condition, *Fs* < 1.

The fact that no effect was observed in the alone condition is also relevant. Although no effect of threat and challenge was expected in the alone condition because of the lack of task engagement, it could be that participants reacted differently to strong and weak arguments, especially those in threat conditions. The fact that they did not suggests that they were not elaborating the message content. In some way, the lack of task engagement produces superficial processing, suggesting a low level of motivation.

These results replicate the effect observed in Experiment 1, supporting the hypothesis that challenge decreases the probability of analytic processing. In addition, they clarify that the activation pattern is dependent upon task engagement, which supposedly promotes a moderate elaborative condition within the dual processing approaches (Petty and Cacioppo, 1986).

### META-ANALYTIC SUMMARY

A meta-analytic summary analysis was conducted to determine the magnitude and consistency of the effect sizes that sustain the two main arguments presented in these experiments. Statistical tests of the overall effect size and the homogeneity (consistency) of those effects were based on Cohen’s *d*. Calculations were aided by ESCI, a program designed for meta-analytic statistics (Cumming, 2012).

The first argument was that our non-demanding and demanding conditions were associated with motivational states of challenge and threat. This association predicts differences both in CO and TPR measures that were clearly found in Experiment 1 but not in Experiment 2 for CO. Converting the F statistics into Cohen’s *d*s, we found that the effect sizes representing the CO effects were *d*= 0.59 (Experiment 1) and *d*= 0.56 (Experiment 2). The overall effect size (*d*= 0.56) was significantly different from zero, *z*= 5.375, *p*< 0.001, and the effects were statistically consistent, *Q*(1) = 1.197, *p*= 0.881. Additionally, by converting the F statistics into *d*s, we found that the effect sizes representing the TPR effects were *d*= 0.66 (Experiment 1) and *d*= 0.65 (Experiment 2). The overall effect size (*d*= 0.645) was significantly different from zero, *z*= 6.00, *p*< 0.001, and the effects were statistically consistent, *Q*(1) = 0.002, *p*= 0.962. These results confirm that demanding and non-demanding conditions were associated with a different pattern of CO and TPR reactions, which provides robust data supporting our claims.

The second and main argument was that our demanding and non-demanding conditions engaged participants in more superficial versus deeper processing, as indicated by similar or different reactions to strong and weak arguments. Converting the t statistics of the relevant contrasts into *d*s, the size of the effect of argument’s quality is *d* = 0.31 for the challenge conditions and *d*= 0.52 for the threat condition (Experiment 1) and *d*= 0.46 for the challenge condition and *d*= 0.60 for the threat condition (Experiment 2- presence condition). For the threat condition, the overall effect size (*d*= 0.45) was significantly different from zero, *z*= 1.967, *p*= 0.049, and the effect sizes were statistically consistent, *Q*(1) = 1.324, *p*= 0.252. The effect was not reliable for the challenge condition. In Experiment 1, *d*= -0.30, and in Experiment 2 (presence condition), *d*= 0.62, turning the overall effect size into *d*= 0.146, which is not reliable, *z* = 1.28, *p = 0*.752, and lacks consistency, *Q*(1) = 12.61, *p*< 0.001. Altogether, data from these two experiments allow us to state that there is a reliable and consistent difference among personal states of challenge and threat in how they process strong and weak arguments: whereas participants processed the persuasive message carefully in the threat state, they did not seem to do so in the challenge state.

## GENERAL DISCUSSION

These two experiments provide data that deal with the question of how motivational states of challenge and threat relate to dual processing. This is the first attempt in the BPS model literature to do so. Data obtained in the two experiments indicate that challenge is associated with more superficial processing. Experiment 1 demonstrated that participants who were challenged during a task engaging visual ability game prior to an information processing task were unable to distinguish strong from weak arguments in the latter and did not differ in persuasibility, which supports a connection between challenge and superficial processing. Furthermore, Experiment 2 demonstrates that these results were not dependent on the association of challenge and threat with different levels of task motivation. Experiment 2 clarifies that the threat manipulation was not sufficient by itself to motivate individuals to process the persuasion task more analytically in the alone condition, as indexed by low task engagement.

Together, these results offer new insights on the mechanisms underlying challenge and performance. Findings regarding the BPS model have shown that challenge is associated with better performance on tasks that require the use of relatively automatic processes (e.g., Blascovich et al., 1993, 2004) but is associated with poorer performances on tasks that require more controlled processes (e.g., Hunter, 2001; Feinberg and Aiello, 2009). In the absence of an explanation, we argued that such discrepancies could be attributed to different information processing modes that underlie challenge and threat. Specifically, we argued that because challenge is, by definition, a state in which one evaluates available resources as outweighing task demands (Blascovich, 2008), challenge, compared to threat, is more likely to be associated with superficial processing (e.g., Garcia-Marques and Mackie, 2001, 2007) and improves performance that requires only this level of processing.

Important to this interpretation is what we understand to be the challenge state. Challenge is activated as result of the individual’s evaluation (conscious or unconscious) that he or she has sufficient or greater than necessary resources available to manage a specific task. This evaluation of resource availability was manipulated here by making a visual ability game easier. Because this first task was unrelated to the second task (the persuasion task), participants did not really have more resources for the second task than those who did not experience the relatively easier task. They did not have the opportunity to store a specific response on a first task that was applicable during the second task (e.g., Fazio et al., 1992; Blascovich et al., 1993).

As with studies that manipulated familiarity with either the materials or the task, familiarity was induced from experience, which led individuals to process information superficially (Garcia-Marques and Mackie, 2001; Smith et al., 2006), as does a simple manipulation of fluency (e.g., Reber et al., 2006; Alter et al., 2007; Oppenheimer, 2008, for reviews see Alter and Oppenheimer, 2009). The experience of such fluency may underlie the observed effects; thus, fluency may be one component integrated in the overall experience of challenge-reducing elaborative processing. Other manipulations of the experience of response or resource availability that could control for ease and fluency (if possible) is something that future research should address.

Related to this question is whether the physiological index of challenge is always associated with superficial processing. Although our data suggest this could be the case, the possibility that analytic processing may occur during a challenge state cannot be ruled out. Recall that challenge and threat at their extremes are endpoints on a bipolar continuum. Their activation is determined by an interactive cognitive evaluation process of resources and demands. Via this process, four conditions of a 2 × 2 matrix may result: high resources, low demands; low resources, high demands; high resources, high demands, and low resources and low demands. In our experiments, we examined only the first possibility and observed that information is processed superficially. However, processing in a situation of high resources/high demands is likely different. For example, Claypool et al. (2004) demonstrated that the greater-availability-of-resources effect on superficial processing is moderated by the level of personal involvement with the situation. This outcome suggests that, under certain circumstances, high resources may not always lead to superficial processing. In the long run, it is of great importance to understand the conditions under which any particular combination occurs (e.g., challenge-superficial; threat-superficial; challenge-analytic; threat-analytic).

Directly related to this point and important to the discussion here is why threat was associated with analytic processing. Although some have demonstrated that mood states triggered by threatening environments are associated with analytic processing (e.g., Bohner et al., 1992; Schwarz, 2004), threat here is defined as a condition in which one evaluates fewer resources to cope with the task demands. Thus, one could argue, according to the cognitive and social cognitive literature (Cialdini et al., 1981; De Neys, 2006), that such participants should be resource depleted and therefore unable to process information analytically. However, this is not as simple as it appears. Although some authors have shown that resource depletion impairs analytic processing, others have shown that cutting off some cognitive resources may actually lead to analytic processing (Lavie et al., 2004; Dalton and Lavie, 2012). Our results in the threat condition corroborate those obtained by Lavie et al. (2004) which may be explained because the difficult version of the game only mildly depleted participants or because the experience of low resources/low demands in the 2 × 2 matrix signals a situation where analytic processing is needed. Either way, having all cognitive resources available and accessible, an experience consistent with the challenge definition, does not seem to necessarily mean more analytic processing.

With this paper, we opened the door for new avenues of research within the well-established BPS of challenge and threat. Some of the limitations of our experiments should be addressed in the future in order to better understand the reliability and generalizability of our findings. Regarding the former, we stress the need to increase the sample size in future studies in order to increase power thereby reducing the possibility of false positive results. Regarding the latter, it would be desirable to examine the same dissociation of processes in new domains; for example, how challenge increases superficial responses in other types of tasks such as decision making and stereotype tasks. In addition, the impact that the presence of others seems to have on engaging individuals in current activities should be clarified in order to understand if features of the confederate (e.g., age and gender) have a role. Nevertheless the findings obtained in this paper direct us to the possibility that the cognitive dimensions of challenge and threat and their implications on how we think and behave are far more complex than previous research has shown. By doing that, we have identified new and important bridges between motivational and cognitive effects (and literature) that traditionally have been kept apart. Here, we pictured part of this complex puzzle. Future research will be pivotal in addressing the questions raised.

## Conflict of Interest Statement

The authors declare that the research was conducted in the absence of any commercial or financial relationships that could be construed as a potential conflict of interest.

## Footnotes

1 The procedure was approved by the University ethics committee.

2 Although the message quality did not have any effects on CO (Fs < 1), it qualified the effect of the game version on TPR, *F*(1, 48) = 4.91, *p*= 0.03, η^2^ = 0.17. Whereas for those in the demanding condition the strong message, in comparison with the weak one, increased TPR values (*M* strong = 184.27, SD = 89.33 vs. *M* weak = -0.25, SD = 96.49), for those in the non-demanding condition, it decreased them (*M* strong = -206.15, SD = 96.49 vs. *M* weak = 21.54, SD = 89.33). The main effect of game version on TPR was not significant, *F* < 1.
