# Supplementary material for: Challenge and threat motivation: effects on superficial and elaborative information processing
Source: Front Psychol. 2014 Oct 14;5:1170. doi: 10.3389/fpsyg.2014.01170 (PMC4196581; doi:10.3389/fpsyg.2014.01170)
Supplement: Supplementary file 1 [file Data_Sheet_1.DOCX]

APPENDIX

**Visual ability game**

The goal is to observe, on the computer screen, for 3 seconds several pairs of geometrical shapes of different sizes, compare their length and then decide which one of the two was bigger.

Instructions: *After each trial and before the beginning of a new one, the computer will rapidly prompt you with the word "next" or the words "non-demanding” or “demanding” inform you that you will see a standard, easier or difficult trial. Thus, you have to pay attention to which word will pop up on the screen and be prepared to overcome every trial. When you see the word, please focus and get ready because a trial will start soon. To respond, use the computer mouse. If you think the shape presented on the left is bigger, click on a box with an L (L for Left) that appears on the screen (below the geometrical shapes). If you think the shape on the right was bigger, click on the box with an R (R for Right).*

| Non-demanding (example) | Demanding (example) |
| --- | --- |
| 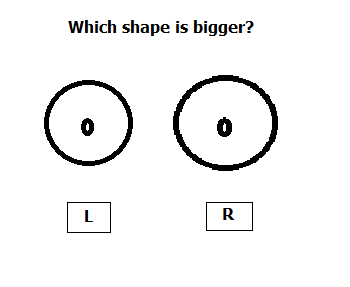 | 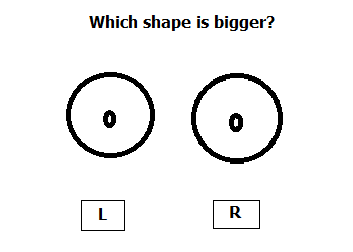 |
